# *Salmonella enterica* Serotype Enteritidis in French Polynesia, South Pacific, 2008–2013

**DOI:** 10.3201/eid2106.141103

**Published:** 2015-06

**Authors:** Simon Le Hello, Fiona Maillard, Henri-Pierre Mallet, Elise Daudens, Marc Levy, Valérie Roy, Philippe Branaa, Sophie Bertrand, Laetitia Fabre, François-Xavier Weill

**Affiliations:** Institut Pasteur, Paris, France (S. Le Hello, F. Maillard, L. Fabre, F.-X. Weill);; Direction de la Santé de la Polynésie Française, Papeete, Tahiti, French Polynesia (H.-P. Mallet, E. Daudens);; Centre Hospitalier de Polynésie Française, Papeete (M. Levy);; Service du Développement Rural, Papeete (V. Roy);; Institut Louis Malarde, Papeete (P. Branaa);; Scientific Institute of Public Health, Brussels, Belgium (S. Bertrand)

**Keywords:** *Salmonella enterica* serotype Enteritidis, CRISPR, MLVA, laying hen, French Polynesia, South Pacific, bacteria, salmonellae

## Abstract

Outbreaks of *Salmonella enterica* serotype Enteritidis infections associated with eggs occurred in French Polynesia during 2008–2013. Molecular analysis of isolates by using clustered regularly interspaced short palindromic repeat polymorphisms and multilocus variable-number tandem-repeat analysis was performed. This subtyping made defining the epidemic strain, finding the source, and decontaminating affected poultry flocks possible.

Over the past 2 decades, the incidence of *Salmonella enterica* serotype Enteritidis infections in humans has increased dramatically in all industrialized countries, with contaminated eggs the major source of infection (*1**,**2*). Despite a substantial decrease in outbreaks caused by this bacterium since the beginning of the 2000s, in particular in Europe due to the introduction of various control measures, *Salmonella* Enteritidis remains a major foodborne pathogen causing considerable human disease and high economic costs (*3**,**5*).

Different phenotypic and genotypic methods have been used to subtype *Salmonella* Enteritidis, including techniques such as phage typing and pulsed-field gel electrophoresis (PFGE). Results suggest the existence of major worldwide clones of *Salmonella* Enteritidis, of which most strains belong to phage type (PT) 4, followed by PT8 and PT1 (*1**,**6*). Recently, new methods such as standardized multilocus variable-number tandem-repeat analysis (MLVA) (*7*) and clustered regularly interspaced short palindromic repeats (CRISPR) typing (*8**,**9*) have been developed to subtype genetically homogeneous serotypes of *Salmonella*, in particular Enteritidis.

We report successive outbreaks of *Salmonella* Enteritidis in French Polynesia, South Pacific. To identify the source and determine the molecular subtypes of *Salmonella* Enteritidis strains that are circulating, we performed a comprehensive molecular and epidemiologic study on human and nonhuman strains isolated in Tahiti during 2008–2013.

## The Study

Six cases of foodborne infection caused by *Salmonella* Enteritidis occurred on the island of Tahiti in October 2011, alerting public health authorities to an abnormal increase of these infections in humans. Epidemiologic and microbiological investigations confirmed that a tuna dish prepared with contaminated raw eggs was the food vehicle. Cases of *Salmonella* Enteritidis infection in Tahiti began to increase in July 2011, peaked in December 2011, and returned to baseline in April 2012; a total of 62 laboratory-confirmed cases occurred (Figure). A resurgence of 15 cases was registered during September–December 2012. Epidemiologic investigation by public health authorities revealed 20 clusters of cases (with a total of 54 cases) associated with the consumption of uncooked eggs produced by local layer farms. During November 2011–December 2012, a survey of 17 local poultry farms indicated the presence of *Salmonella* Enteritidis in 14 (1.9%) of 739 samples: 0 of 6 from drinking water sources, 0 of 15 from poultry feed, 3 (1.9%) of 155 from dust, 6 (1.5%) of 391 from feces, and 5 (2.9%) of 172 from eggs. The samples that tested positive were from 5 laying-hen houses on 2 farms that produce 3,000,000 eggs per year (70% of the local production).

**Figure:**
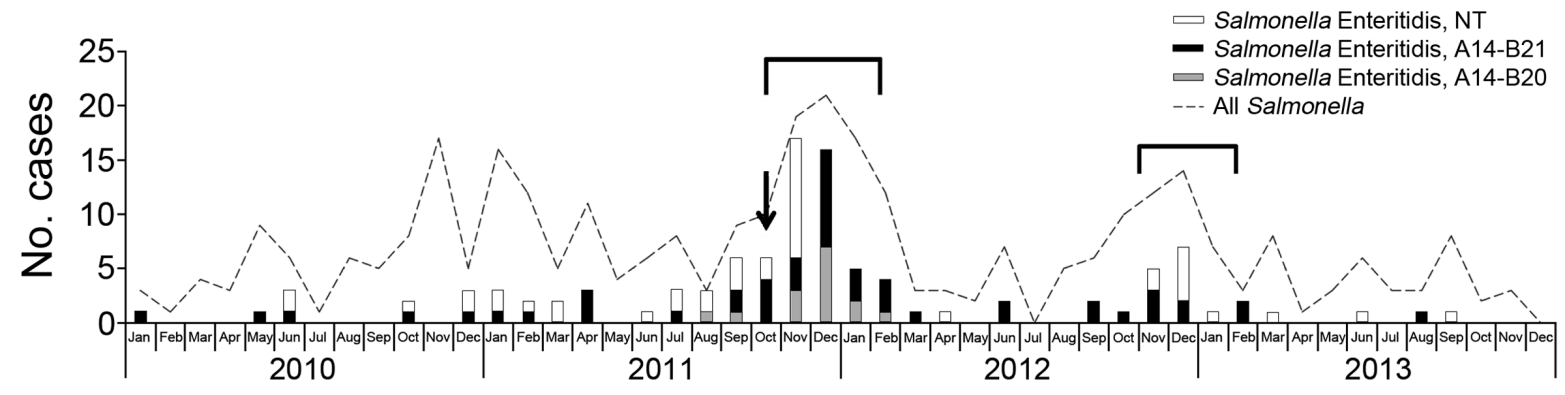
Number of confirmed cases of human infection with *Salmonella enterica* serotype Enteritidis per month and distribution of clustered regularly interspaced short palindromic repeats types, French Polynesia, 2010–2013. Arrow indicates when infections associated with tuna dish prepared with contaminated eggs occurred; brackets indicate periods of laying hen slaughters. NT, not typed.

A total of 112 *Salmonella* Enteritidis strains isolated in French Polynesia were sent to the Centre National de Référence des *Escherichia coli*, *Shigella*, et *Salmonella* for further analysis. During January 2008–August 2013, a total of 111 strains were isolated (96 from humans, 1 from the tuna dish, and 14 from laying hens); in November 2014, 1 strain was isolated from an imported chicken product from the United States. All but 3 *Salmonella* Enteritidis strains were susceptible to all antimicrobial drugs tested (*10*); the remaining 3 showed single-drug resistance to amoxicillin (data not shown). 

Analysis by PulseNet (http://www.cdc.gov/pulsenet/pathogens/index.html) standardized *Xba*I PFGE showed a similar common profile, named JEGX01.0004 in a previous study (*11*), in 46 of 47 selected strains from Tahiti (Tables 1, 2). Phage typing revealed mostly 2 types, PT8 (n = 8) and PT13a (n = 4), for strains with the JEGX01.0004 profile. MLVA typing (*7*) on a subset of 60 strains showed main diversity in the SENTR4 and SENTR5 loci in isolates with the JEGX01.0004 PFGE profile. MLVA types 2-10-8-5-2 and 2-10-8-6-2 dominated in strains isolated from humans and laying hens. The CRISPR1 and CRISPR2 polymorphisms in 83 selected strains were studied by PCR amplification and sequencing as described elsewhere (*9*). The spacer content was determined by submitting the DNA sequences to the Institut Pasteur CRISPR database for *Salmonella* (http://www.pasteur.fr/recherche/genopole/PF8/crispr/CRISPRDB).

**Table 1:** CRISPR-type characteristics of 67 *Salmonella enterica* serotype Enteritidis clinical isolates from French Polynesia, 2008–2013, compared with major examples from the Institut Pasteur database*

Country and period of isolation	No. isolates	Major PFGE types (no.)	Phage types available (no.)	CRISPR type allele1-allele2	MLVA type (no.)†
French Polynesia					
2008 Jan–2013 Aug	52	JEGX01.0004 (13)	PT8 (1), PT13a (2)	A14-B21	2-10-8-5-2 (20). 2-10-8-5-1 (1), 2-11-8-5-2 (6), 2-9-8-5-2 (5), 2-12-9-5-2 (1), 2-12-5-5-2 (1)
2011 Aug–2012 Feb	15	JEGX01.0004 (4)	PT8 (2)	A14-B20	2-10-8-6-2 (15)
France					
1957–2013	83	XEN-001 (57)	PT4 (45), PT1 (10), PT6 (6), PT21 (3), PT14b (1), PT22 (1), PT24 (1), PT34 (1), PT35 (1), PT44 (1)	A6-B7	3-11-5-4-1 (6), 3-11-5-6-1 (1), 3-10-5-4-2 (1), 3-10-5-4-1 (1)
2002	10	XEN-001 (10)	PT4 (6), PT35 (3), PT6a (1)	A8-B7	
1956–2014	8	XEN-001 (6)	PT4 (6)	A7-B7	2-9-4-5-1 (1), 1-8-9-4-1 (4)
1920–2001	7	XEN-001 (4) XEN-008 (2)	PT4 (6) PT6a (1)	A10-B7	
1956–2011	54	JEGX01.0004 (42)	PT8 (28), PT14b (12), PT13a (1), PT22 (1)	A14-B6	2-12-7-5-1 (1)

*All available CRISPR-types, and the spacer content of each, are described in online Technical Appendix 1 (http://wwwnc.cdc.gov/EID/article/21/6/14-1103-Techapp1.xlsx). CRISPR, clustered regularly interspaced short palindromic repeats. †SENTR7-SENTR5-SENTR6-SENTR4-SE3.

**Table 2:** Epidemiologic data, antimicrobial susceptibility patterns, *Xba*I PFGE types, phage types, MLVA types, and CRISPR types of nonhuman *Salmonella enterica* Enteritidis serotype isolates from French Polynesia, 2011–2014*

Period of isolation	Origin of sample	Sample type (no.)	No. isolates	Antimicrobial resistance profile (no.)	PFGE types (no.)	Phage types (no.)	CRISPR types, allele1-allele2 (no.)	MLVA type (no.)†
2011 Oct 25	Restaurant	Tuna dish with raw eggs	1	Susceptible	JEGX01.0004		A14-B20	2-10-8-6-2
2011 Nov–Jan 2012	Farm A	Egg (5), feces (1)	6	Susceptible (6)	JEGX01.0004 (5), XEN-033 (1)	8 (2), 23 (1)	A14-B21 (5), A14-B20 (1)	2-10-8-5-2 (4), 2-11-8-5-2 (1), 2-10-8-6-2 (1)
2011 Jan–2012 Dec	Farm B	Feces (5), dust (3)	8	Susceptible (8)	JEGX01.0004 (8)	8 (3), 13a (2)	A14-B21 (8)	2-10-8-5-2 (3), 2-11-8-5-2 (1), 2-9-8-5-2 (4)
2014 Nov	Imported chicken product	Legs–official control	1	NP	NP	NP	A14-B21	NP
*The spacer content of each CRISPR-type is described in online Technical Appendix 1 (http://wwwnc.cdc.gov/EID/article/21/6/14-1103-Techapp1.xlsx). CRISPR, clustered regularly interspaced short palindromic repeats; MLVA, multilocus variable-number tandem-repeat analysis; NP, not performed; PFGE, pulsed-field gel electrophoresis. †SENTR7-SENTR5-SENTR6-SENTR4-SE3.								

The 83 strains from French Polynesia had the same CRISPR1 allele (A14) but 2 different CRISPR2 alleles (B20 or B21), differing by the presence of a single spacer, EntB9 (Technical Appendix 1; Technical Appendix 2). Both CRISPR2 alleles contained a triplication of the EntB8 spacer, which had not been observed in our database (194 *Salmonella* Enteritidis strains from France and Europe during 1920–2014) (*9*). However, this particular A14-B21 CRISPR profile is displayed by 37 *Salmonella* Enteritidis genomes deposited in the GenBank public database and originating in poultry or humans from North America (*8**,**11**,**12*) (Technical Appendix 3).

Locally, in the month after the outbreak associated with consumption of the tuna dish, different control measures were implemented, depending on whether eggs were contaminated. Workers at farm A, where eggs were contaminated by both A14-B20 and A14-B21 strains, slaughtered laying hens. At farm B, where contamination was revealed only by sampling dust and feces (with only an A14-B21 CRISPR profile for *Salmonella* Enteritidis), minimal sanitary policies were implemented (i.e., thermically treating eggs, disinfecting laying houses). Consequently, the incidence of human *Salmonella* Enteritidis infections has declined markedly in Tahiti. The reisolation of A14-B21 *Salmonella* Enteritidis strains from humans and farm B at the end of 2012 necessitated stronger measures, including slaughtering more laying hens. In total, 120,000 hens were slaughtered, representing 50% of the stock in Tahiti, which caused an egg-production deficit. After this outbreak ended in 2013, production levels returned to normal. Furthermore, controls on imported chicken products have begun in French Polynesia, and in November 2014, a frozen chicken product from the United States tested positive for *Salmonella* Enteritidis A14-B21. Given that the poultry sector has been importing eggs and laying hens from North America for decades, that the A14-B21 CRISPR profile is prevalent in *Salmonella* Enteritidis genomes from North America, and that a A14-B21 *Salmonella* Enteritidis strain has recently been isolated from imported poultry from the United States since the implementation of control on imported poultry products and animals, it is likely that the epidemic *Salmonella* Enteritidis strain that was circulating in French Polynesia was imported from North America before 2008.

## Conclusions

When analyzed by classical subtyping methods, the *Salmonella* Enteritidis strains from French Polynesia displayed a very common and global profile, JEGX01.0004 PFGE type, PT8, and pansusceptibility to antimicrobial agents. Because of this, we used a combination of methods, such as CRISPR typing and MLVA, to more precisely define the epidemic strain and confirm that 2 local poultry farms were the source of the increase in human cases in Tahiti during July 2011–April 2012. By applying minimal to maximal control measures, depending on the CRISPR profile, and by sampling these flocks regularly, it became possible to follow and readjust the efficacy of the different control measures taken by the 2 layer farms. We also demonstrated that the epidemic strain has been circulating in French Polynesia since at least 2008 and was probably imported from North America but has not been associated with human cases since 2014.

Given the signatures offered by the polymorphism of the 2 CRISPR loci in our study and in previous works (*8*, *9**,**13*), we are convinced that CRISPR DNA targets might be very helpful for subtyping *Salmonella*, including serotype Enteritidis. Furthermore, because the CRISPR spacer content can be extracted easily from short-read DNA sequences, in contrast to MLVA loci, it could be used to define particular *Salmonella* Enteritidis strains together with, or as an alternative to, core genome single nucleotide polymorphisms when whole-genome sequencing for foodborne pathogen surveillance and investigation are implemented in public health and veterinary laboratories (*14*).

**Technical Appendix 1.** Clustered regularly interspaced short palindromic repeats pattern distribution described by the Centre National de Référence des *Escherichia coli*, *Shigella*, et *Salmonella*.

**Technical Appendix 2.** Accession numbers for clustered regularly interspaced short palindromic repeats sequences of *Salmonella* strains tested in the present study.

**Technical Appendix 3.** Clustered regularly interspaced short palindromic repeats profile displayed by 37 *Salmonella enterica* Enteritidis genomes deposited in the GenBank public database
